# Increased survival with the combination of stereotactic radiosurgery and gefitinib for non-small cell lung cancer brain metastasis patients: a nationwide study in Taiwan

**DOI:** 10.1186/s13014-015-0431-7

**Published:** 2015-06-06

**Authors:** Ching-Heng Lin, Kuo-Hsuan Hsu, Shih-Ni Chang, Hsi-Kai Tsou, Jason Sheehan, Meei-Ling Sheu, Hung-Chuan Pan

**Affiliations:** Department of Medical Research, Taichung Veterans General Hospital, Taichung, Taiwan; Department of Chest Medicine, Taichung Veterans General Hospital, Taichung, Taiwan; Functional Neurosurgery Division, Neurosurgical Institute, Taichung Veterans General Hospital, 1650 Taiwan Boulevard Sec.4, 40705 Taichung, Taiwan; Department of Neurosurgery, University of Virginia, Charlottesville, VA USA; Institute of Biomedical Sciences, National Chung-Hsing University, Taichung, Taiwan; Faculty of Medicine, School of Medicine, National Yang-Ming University, Taipei, Taiwan

**Keywords:** IRESSA, Gamma knife, Lung cancer, Brain irradiation

## Abstract

**Purpose:**

Whole brain irradiation (WBRT) either with or without resection has historically been the treatment for brain metastases from non-small cell lung cancer (NSCLC). The effect of gamma knife (GK) radiosurgery, chemotherapy, or the combination remains incompletely defined. In this study, we assessed the outcome of brain metastases from non-small cell lung cancer treated by WBRT followed by GK, gefitinib, or the combination of GK and gefitinib.

**Material and methods:**

We retrieved the records of NSCLC patients with brain metastases from the National Health Insurance Research Database (NHIRD) of Taiwan from 2004 to 2010. WBRT either with or without resection was the first line treatment for nearly all patients. The decision to add GK and/or gefitinib treatment was at the discretion of the treating physician and based upon a patient’s medical records and imaging data. These patients were classified into four groups including WBRT, WBRT + gefitinib, WBRT + GK, WBRT + gefitinib + GK. These data was evaluated for difference in survival and factors that portended an extended survival from the time of brain metastasis diagnosis.

**Results:**

Of the 60194 patients with newly diagnosed NSCLC, 23874 (39.6 %) developed brain metastases. The distribution of patients for the groups was WBRT for 20241, WBRT + gefitinib for 3379, WBRT + GK for 155, and WBRT+ gefitinib + GK for 99 patients. The median survival for the time of brain metastasis diagnosis for WBRT, WBRT+ gefitinib, WBRT+ GK, WBRT+ gefitinib + GK groups was 0.53, 1.01, 1.46, and 2.25 years, respectively (*p* < 0.0001). The hazard ratio (95 % CI) for survival was 1, 0.56, 0.43, and 0.40, respectively (*p* < 0.001). The adjusted hazard ratio (95 % CI) by age, sex and Charlson comorbidity index (CCI) was 1, 0.73, 0.49, and 0.42, respectively (*p* < 0.001).

**Conclusion:**

Patients with brain metastases from NSCLC receiving GK or gefitinib demonstrated extended survival. The improved survival seen with GK and gefitinib suggests a survival benefit in selected patients receiving the combined treatment. Further Phase II study should be conducted to assessment these influence.

## Introduction

Lung cancer harbored the highest incidence of brain metastasis in relation to all malignancies. Approximately 40 % of all patients with non-small cell lung cancer (NSCLC) will develop brain metastasis during the course of their disease [1]. Even with treatment, the prognosis for these patients remains poor with a median survival of 7 months. Traditionally, WBRT is the first line treatment, but should be tailored according to the patients’ condition, the number and size of metastases, etc. [2].

GK can be used to treat multiple metastases during the same procedure and permits treatment of deep seated lesion considered surgical inaccessible [3–6]. Subset analysis of a randomized trial demonstrated improved survival with the addition of SRS to WBRT in patients with single brain metastases and in patients younger than 65 with good performance status, controlled primary tumor, and no extracranial metastases compared to those receiving WBRT alone [7]. Other randomized trials comparing SRS alone to WBRT and SRS combined have a reduction in intracranial relapse and reduced rate of neurological death with the addition of WBRT [8, 9]. In contrast, another study showed worsened overall survival and neurocognition at 4 months after WBRT compared to treatment with SRS alone [10]. Therefore, National Comprehensive Cancer Network (NCCN) guidelines recommend consideration of SRS for patients with 1–3 brain metastases with newly diagnosed or stable systemic disease or for those with reasonable systemic treatment options.

In two randomized phase II trials, the efficacy of gefitinib showed encouraging activity, in term of the objective response rate and clinical benefit with symptomatic improvement in patients with advanced NSCLC after failure of one or two previous chemotherapy regimens [11, 12]. Several groups reported that a substantial percentage of NSCLC tumors getting objective response when treated with epidermal growth factor receptor (EGFR) tyrosine kinase inhibitors (TKI) harbor activating somatic mutation in the EGRF gene including in frame deletion or amino-acid substitution clustered around ATP- binding pocket of EGFR tyrosine kinase domain (in exons 18, 19, and 21) [13–16]. Limited data existed for the responsiveness of brain metastases to EGFR inhibitor gefitinib [17–21]. In the large prospective series study, as with extracranial disease, the response of brain metastases to EGFR inhibitors seems to depend upon the presence of an EGFR mutation [22].

The combination of EGFR TKI and radiation has enhanced effects for inhibition of proliferative and antiapopotic signaling pathways downstream of EGFR in cancer cell lines [23, 24]. A combination treatment of WBRT and gefitinib achieved significant tumor response and longer median survival as well as little toxicity in a Chinese population [25]. However, debate persists regarding the role of radiosurgery or radiation therapy in combined with gefitinib in brain metastasis from NSCLC patients. In this study, we retrieved data from the NHIRD bank and stratified the NSCLC patients with brain metastasis to four groups as follows: (1) WBRT alone; (2) WBRT+ gefitinib; (3) WBRT+ GK; and (4) WBRT+ gefitinib + GK. We then evaluated for difference in survival between the groups and prognostic factors related to improved survival from the time of brain metastasis diagnosis. We hope to discern the utility of GK or gefitinib in NSCLC patients with brain metastasis after WBRT.

## Material and methods

### Data sources

Since 1995, Taiwan established its state-run National Health Insurance (NHI) program, which covers more than 99 % of the island’s population and has contracted with 97 % of the hospitals. Data analyzed in this study were retrieved from the Taiwan National Health Insurance Research Database (NHIRD), which is managed by the Taiwan National Health Research Institute (NHRI). Details of this population-based database have been described previously. Diagnoses were coded with the International Codes of Disease 9th Edition Clinical Modification (ICD-9-CM).

### Study population

The study subjects were retrieved the newly defined NSCLC with brain metastases from the NHIRD between January 1, 2004, and December 31, 2010. The diagnostic accuracy of NSCLC was confirmed by inclusion in the Registry for Catastrophic Illness Patient Database (RCIPD), a subpart of the NHIRD. Histological confirmation of NSCLC is required for patients to be registered in the RCIPD.

There were a total of 60149 patients diagnosed as NSCLC and 23874 (39.6 %) with brain metastasis in the study cohort were divided into the aforementioned four cohorts. The WBRT was comprised of a radiation dosage of 24 to 30 Gy in 8 to 10 fractions. As first line treatment in Taiwan, WBRT either with or without craniotomy was delivered. In general, GK was utilized if the following criteria were met: number of lesions <3, individual size of tumor <20 cc, and KPS > 70. Gefitinib was used in patients if there was evidence of an EGFR mutation. Gamma knife or gefitinib could also be utilized at the discretion of the treating neurosurgeon and medical oncologist, and in such circumstances, the decision was based on the patient’s medical records and imaging data contained within the central bureau of Taiwan National Health Insurance Institute. Thus, patients were allocated into four treatment groups: WBRT (*n* = 20241), WBRT + gefitinib (*n* = 3379), WBRT+ GK (*n* = 155), and WBRT+ gefitinib + GK (*n* = 99).

The index date for each subject was the first treatment date. Study end-point were defined as the patients were follow-up from index date until death, withdraw of the database or the end of 2010.

The survival time, age, sex, brain surgery, and Charlson comorbidity index were obtained for statistical analysis.

### EGFR mutation analysis

Mutation analysis was conducted in an institutional core facility regulated by the Clinical Laboratory Improvement Amendments as below. The analysis of EGFR gene mutations were conducted in paraffin-embedded tissue sections from the primary lung cancer. Tumor tissue was scraped from the glass side under direct visualization or under a dissecting microscope. DNA was extracted with a QIAmp DNA Mini Kit (Qiagen, Valencia, CA). EGFR mutations were performed by DNA sequencing as follows. *EGFR* exons 18 to 21 were sequenced with a BigDye Terminator v3.1 Cycle Sequencing Kit (Applied Biosystems, Foster City, CA) after nested polymerase chain reaction as previously described [14].

### Gamma knife technique

All patient were treated with the Leksell gamma knife; Gamma Knife units utilized included a model B (*n* = 1), C (*n* = 1), 4C (*n* = 3) and Perfexion (*n* = 2) (Elekta AB). All GK was delivered via a multidisciplinary approaching and the team consisted of a neurosurgeon, neuroradiologist, radiation oncology and medical physicist. The GK technique followed a treatment guideline developed by the Taiwan neurosurgical association. The dosage prescription and number of lesions treated were individually determined by expertise teams.

### Statistical analysis

Distributions of the four groups according to age, gender and clinical characteristics were examined using chi-squared tests. For estimating the risk of mortality in patients with different treatment types, we performed survival analysis using the Kaplan-Meier method, with significance based on the log-rank test. Multivariate Cox proportional hazards regression models were used to explore the relation between treatment modality and mortality, adjusted for age, gender and CCI. The crude and adjusted hazard ratios with 95 % confidence interval (CI) were calculated. The proportional hazards assumption was tested graphically and by including the interaction of time with each covariate. A two-tailed p value of <0.05 was considered statistically significant. All statistical analyses were performed with SAS (version 9.2; SAS Institute, Cary, NC).

## Results

### Patient and group attributes

The characteristics of patients stratified by age, sex, number of CCI, and status of decease was shown in Table 1. There was a male predominance in the overall group with 66.9 % (15978/23847) as well as in subgroups including the WBRT group with 71.2 % (14404/20241) and the WBRT + GK group with 61.9 %(96/155), respectively. The age distribution of ≧65 and <65 was 55.4 % vs 44.7 % in the whole population. However, patients < 65 years old comprised 61.2 % of WBRT + gefitinib group, 72.9 % of the WBRT + GK group, and 78.8 % of the WBRT + GK+ gefitinib group, respectively. A Charlson comorbidity index (CCI) of 2 was seen in 15207 of 23874 (63.7 %) of patient in the overall population. In the WBRT group, 13362 of 20241 (66 %) patients had CCI of 2 and 16.4 % of the patients had CCI ≧6. However, in WBRT+ gefitinib group, there were 49.9 % and 35.5 % in CCI of 2 and ≧6, respectively; in WBRT+ GK group, there were 67.1 % and 21.9 % in CCI of 2 and ≧6, respectively; in WBRT + GK+ gefitinib group, there were 56.6 % and 36.4 % in CCI of 2 and ≧6, respectively.

**Table 1:** Characteristics of NSCLC patient with brain metastases in 2004-2010

	WBRT n = 20241	WBRT+ gefitinib n = 3379	WBRT + GK n = 155	WBRT+ gefitinib + GK n = 99	Total n = 23874	*p* ^*^
Variables	n (%)	n (%)	n (%)	n (%)	n (%)	
Sex						<0.0001
Women	5837 (28.8)	1948 (57.6)	59 (38.1)	52 (52.5)	7896 (33.1)	
Men	14404 (71.2)	1431 (42.4)	96 (61.9)	47 (47.5)	15978 (66.9)	
Age, years						<0.0001
<65	8400 (41.5)	2068 (61.2)	113 (72.9)	78 (78.8)	10659 (44.7)	
≧65	11841 (58.5)	1311 (38.8)	42 (27.1)	21 (21.2)	13215 (55.4)	
CCI						<0.0001
2	13362 (66.0)	1685 (49.9)	104 (67.1)	56 (56.6)	15207 (63.7)	
3	2068 (10.2)	313 (9.3)	8 (5.2)	4 (4.0)	2393 (10.0)	
4	1050 (5.2)	127 (3.8)	7 (4.5)	2 (2.0)	1186 (5.0)	
5	450 (2.2)	53 (1.6)	2 (1.3)	1 (1.0)	506 (2.1)	
≧6	3311 (16.4)	1201 (35.5)	34 (21.9)	36 (36.4)	4582 (19.2)	
Deceased						<0.0001
No	7505 (37.1)	1460 (43.2)	86 (55.5)	47 (47.5)	9098 (38.1)	
Yes	12736 (62.9)	1919 (56.8)	69 (44.5)	52 (52.5)	14776 (61.9)	

^*^chi-square test; CCI: see text

### Patient survival

The median and minimum follow up time in WBRT were 0.53 and 0.03 years, respectively. The median and minimum follow up time in WBRT + gefitinib were 1.01 and 0.05 years, respectively. The median and minimum follow up time in WBRT + GK were 1.46 and 0.074 years, respectively. The median and minimum follow up time in WBRT+ gefitinib + GK were 2.25 and 0.151 years, respectively.

At last follow up, 61.9 % of patients were deceased in the entire study population. Those deceased comprised 62.9 % of the WBRT alone group, 56.8 % of the WBRT+ gefitinib group, 44.5 % of the WBRT + GK group, and 52.5 % of the WBRT+ gefitinib + GK group. The characteristic of patients was subcategorized by gender, and the data showed very similar demographics compared to the whole series.

The hazard ratio of death associated with different treatment modality is detailed in Table 2. The median survival for WBRT, WBRT+ gefitinib, WBRT+ GK, WBRT+ gefitinib + GK was 0.53, 1.01, 1.46, and 2.25 years, respectively (*p* < 0.0001). The crude hazard ratio (95 % CI) of WBRT+ gefitinib, WBRT+ GK, WBRT+ gefitinib + GK related to WBRT were 0.56 (0.62-0.68) (*p* < 0.0001), 0.43 (0.34-0.54) (*p* < 0.0001), and 0.40(0.30-0.52) (*p* < 0.0001). The adjust hazard ratio by age, sex, and CCI were 0.73 (0.70-0.78) (*p* < 0.0001), 0.49(0.36-0.66) (*p* < 0.0001), and 0.42(0.30-0.59) (*p* < 0.0001). In addition, if the hazard ratio was only adjusted by sex, the statistical data did not reveal a significant difference between male and female groups.

**Table 2:** Hazard ratios (HR) with 95 % CI for the association between death and treatment type

Treatment type	Median SY^a^	cHR (95 % CI)	aHR (95 % CI)
WBRT	0.53	1.00 (reference)	1.00 (reference)
WBRT+ gefitinib	1.01	0.56 (0.62-0.68)*	0.73 (0.70-0.78)*
WBRT + GK	1.46	0.43 (0.34-0.54)*	0.49 (0.36-0.66)*
WBRT+ gefitinib + GK	2.25	0.40 (0.30-0.52)*	0.42 (0.30-0.59)*

cHR: crude HR; aHR: adjust for age, sex, CCI

^a^Median SY, median survival year

*p < 0.0001

The rate of patients who underwent brain surgery were 2.1 % of WBRT group, 3.3 % of WBRT + gefitinib group, 12.9 % of WBRT + GK group, and 13.1 % of WBRT+ gefitinib + GK group, respectively. For examining whether “brain surgery” was a confounder of present study outcome, we conducted a multivariate model which adds “brain surgery” as a variable for adjustment (Table 3). The results show no statistically significant differences between two models.

**Table 3:** Hazard ratios (HR) with 95 % CI for the association between death and treatment type

Treatment type	Number	No. of brain surgery (%)	aHR (95 % CI)
WBRT	20241	420 (2.1)	1.00 (reference)
WBRT+ gefitinib	3379	113 (3.3)	0.74 (0.70-0.79)^*^
WBRT + GK	155	20 (12.9)	0.50 (0.37-0.68)^*^
WBRT+ gefitinib + GK	99	13 (13.1)	0.43 (0.31-0.61)^*^

*cHR* crude HR, *aHR* adjust for age, sex, CCI, and brain surgery

^+^Median SY, median survival year

^*^ *p* < 0.0001

**Fig. 1:**
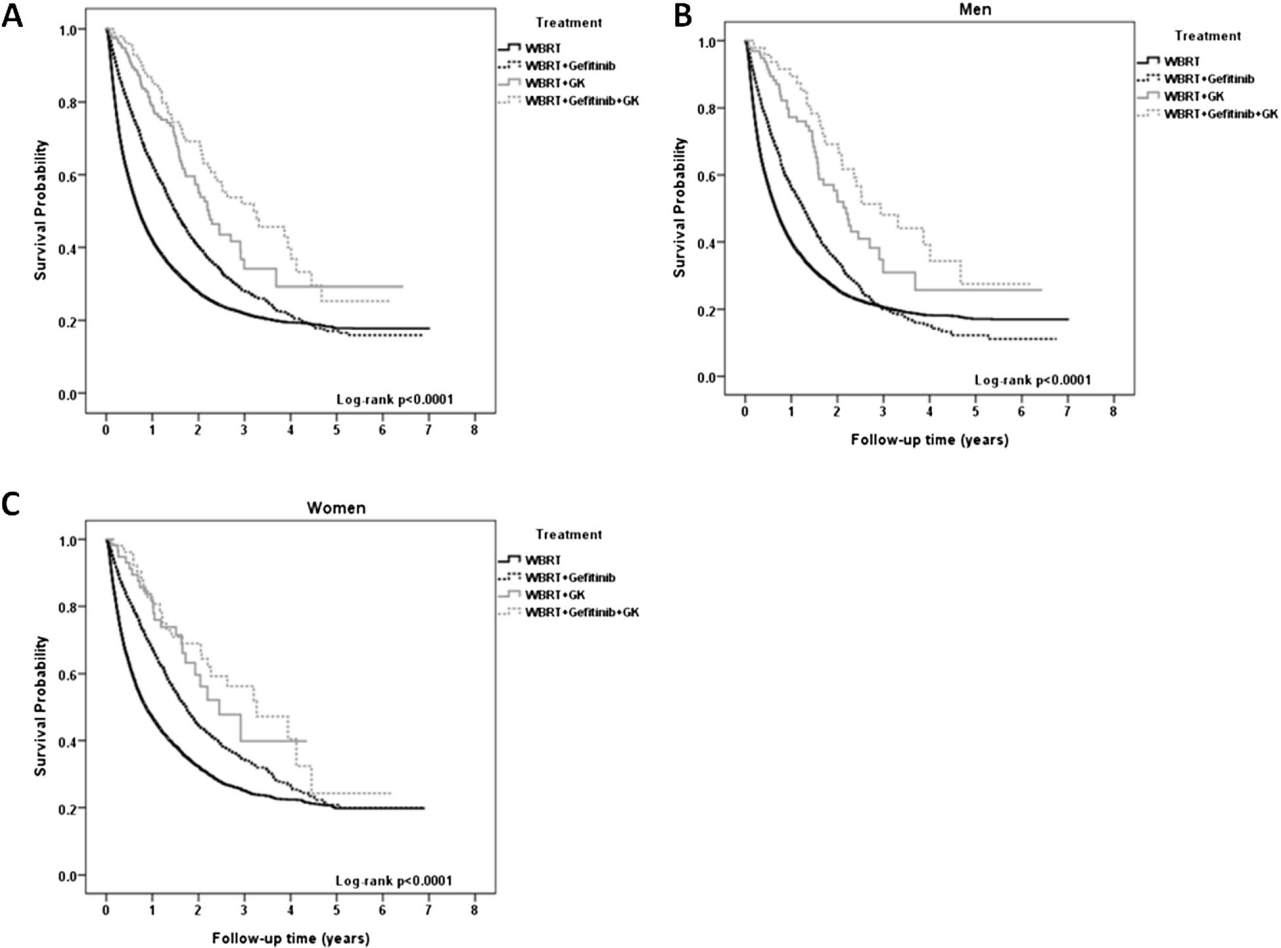
Survival curves in patients with brain metastases from NSCLC for the various treatment regimens. **a** Survival curve for the whole population. **b** Survival cure for male study patients alone. **c** Survival curve for female study patients alone. WBRT, WBRT+ gefitinib, WBRT+ GK, WBRT+GK+ gefitinib: see text

The number of deaths and cumulative death rate by follow-up time are shown in Table 4. The survival curve is demonstrated in Fig. 1a. In Log Rank analysis, there was a statistically significant difference in survival between these four groups (*p* < 0.0001). There was also significantly increased survival between WBRT + GK and WBRT (p < 0.0001), WBRT+ gefitinib and WBRT (*p* < 0.001). These data also demonstrate that WBRT followed by a combination of gefitinib and GK exerted a significantly increased survival as compared to gefitinib or GK alone (*p* < 0.001, *p* < 0.001, respectively). This finding indicates that therapeutic benefit of the GK and gefitinib combination on patient survival. In addition, the survival curves showed no significant difference stratified by sex (Fig. 1b and c).

**Table 4:** Number of death and cumulative death rate by follow-up time

	0-1 year	1-2 years	2-3 years	3-4 years	4-5 years	5-6 years
Treatment type	n (%)	n (%)	n (%)	n (%)	n (%)	n (%)
WBRT	10744 (53.1)	12265 (60.6)	12621 (62.4)	12707 (62.8)	12735 (62.9)	12736 (62.9)
WBRT+ gefitinib	1168 (34.6)	1678 (49.7)	1849 (54.7)	1900 (56.2)	1917 (56.7)	1919 (56.8)
WBRT + GK	30 (19.4)	55 (35.5)	68 (43.9)	69 (44.5)	69 (44.5)	69 (44.5)
WBRT + gefitinib + GK	15 (15.2)	30 (30.3)	43 (43.4)	48 (48.5)	52 (52.5)	52 (52.5)

## Discussion

Brain metastases occur in as many as 47 % of patients with recurrence adenocarcinoma of lung [26]. Even with WBRT or systemic chemotherapy, the outcome of NSCLC patients with brain metastases is still very poor. In this study, we found that addition of gefitinib or GK to WBRT prolonged the median survival of NSCLC patients. Furthermore, the combination of GK and gefitinib to WBRT further improved the overall survival. In part, significant improvements in outcomes of brain metastasis patients are likely to be driven by targeted therapies aimed at specific biological features of cancer subtypes including those with NSCLC as well as breast cancer and melanoma [27, 28].

In a trail of 1692 patients randomly assigned to receive either gefitinib or placebo without any selection according to molecular characteristic, the primary end-point of this study showed no significant difference between groups, neither in overall survival nor among the 812 patients with adenocarcinoma [29]. Another meta-analysis comparing gefitinib to docetaxel showed pooled results that were in line with individual studies, with no significant difference in the overall survival and progression-free survival, and a significant increase in chance of objective response [30–32]. Multiple smaller trials were conducted testing gefitinib as a first line treatment for patients selected with the presence of EGFR mutation. The results of these smaller studies confirmed gefitinib to be generally well tolerated and associated with an objective response rate, progression-free survival and overall survival superior to that expected from traditional chemotherapy regimens [33–38]. It was also evident that a high prevalence of EGFR mutation is more frequent in smokers, woman, and patients with adenocarcinoma [39]. In the current study, the patients undergoing gefitinib treatment meet generally exhibited an EGFR mutation. However, there was no significant difference in median survival by gender for that receiving gefitinib treatment.

Charlson comorbidity index was first developed to predict the risk of mortality using the medical record [40]. The CCI was a better predictor of survival for lung cancer patients treated with surgery compared with individual comorbid condition [41]. It was observed that patients with CCI ≧ 2 had a higher perioperative mortality and death from noncancer causes after lung cancer surgery than patients with index < 2 [42]. In a study to predict survival of lung cancer patients correlated to chronic medical disease, the data showed CCI did not provide predictive validity in lung cancer patient’s survival [43]. In the current study, all of the patients had a CCI scores > 2 and hazard ratio adjusted for survival did not cause a significant alternation compared to the original data. The lack of a significant alteration in survival by CCI in the current study may be explained by the fact that most of the patients had high CCI scores to begin with, and this narrower range resulted in less power for the CCI to effect overall survival.

In recent years, management of brain metastasis from NSCLC has been refined and now includes surgical resection for single brain lesions [44]. The surgery seemed a confounding factor to influence the predicting power in this article. For examining where brain surgery was a confounder factor in this study, we conducted a multivariate model using brain surgery as a variable for adjustment. The data shows no difference between two models.

Gefitinib has a low molecular weight and excellent cell penetration; animal studies have demonstrated a low concentration of 14C-labeled gefitinib in normal rat brain and spinal cord [45] and significant activities against brain tumors in a mouse model [46]. There were some reports showing favorable gefitinib activity fighting brain metastasis from NSCLC [17–22]. Furthermore, the potential beneficial interaction between EGFR inhibition and radiation has been investigated as a major clinical milestone with results from phase III trial in advanced in advanced head and neck cancer patients [47]. The combined treatment of WBRT with EGFR inhibitors in brain metastases from NSCLC resulted in a favorable tumor response and prolonged median survival with little toxicity [25, 48]. In our study, we also found that gefitinib when combined with WBRT prolonged the median survival compared to WBRT alone from 0.53 to 1.01 years; this improvement in survival is comparable to previous reports. Furthermore, the addition of gefitinib with GK+ WBRT improved the median survival to 2.25 years, and it raised the possibility that gefitinib combined with WBRT and radiosurgery can yield a more powerful tumoricidal effect.

Controversy exists regarding the optimal treatment of brain metastases. Randomized trial comparing SRS alone to WBRT and SRS combined have shown conflicting results for patients with 1–4 brain metastases [8]. Additionally, questions about the cost effectiveness have fueled controversy regarding the use of radiosurgery in certain cohorts of brain metastasis patients [49]. NCCN guidelines recommended consideration of SRS for patients with 1–3 brain metastases with newly diagnosed or stable systemic disease or for those patients with reasonable systemic treatment option. In our study, gamma knife radiosurgery was only utilized for treatment in patients with 3 or fewer brain metastases and a KPS > 70. Thus, the findings of longer survival in the WBRT + GK and WBRT + GK+ gefitinib cohorts must be considered in the context of the selection biases of this study.

In 2013, RTOG published a randomized trial of 126 patients treated with WBRT and stereotactic radiosurgery alone versus WBRT and SRS with temozolmide or erlotinib for NSCLC. The results showed no improved survival and possibly deleterious effect with erlotinib [50]. The results seem that erlotinib was a confounder in brain metastases. If we looked into the data comparison, we could find that in our study, those who underwent IRRESSA treatment should have EGFR mutation. In the above study, we did not find that authors stratify the treatment according to expression of EGFR mutation. This might be the reason that there existed the substantial difference between these two studies.

The distributions of patients in four groups don’t seem appropriately balanced. The distribution of patients for the groups was WBRT for 20241, WBRT + IRESSA for 3379, WBRT + GK for 155, and WBRT + IRESSA + GK for 99 patients. There should present a debate that why more physicians/neurosurgery decided to treat those patients with WBRT + IRRESA instead of WBRT + GK. The way of the decision was based on our insurance policy that in those patients with EGFR mutation and brain metastases, the physician jumped to the decision of WBRT combined with IRRESSA. In the patients without ERGR mutation, the WBRT either combined with or without GK was then conducted. Hence, there were more patients treated with WBRT + IRRESA than WBRT + GK.

There were some limitations in our study. First of all, in general, WBRT was the standard treatment in our patients. Gefitinib is persevered for patient with EGFR mutation and SRS for brain lesions less than 3. The better outcome in our combined treatment compared to WBRT alone cohort had the possibility of selection biases. Second, in our favorable outcome group as compared to WBRT alone, the age distribution seemed younger than those in WBRT. There will be a confounding factor in predicting the survival. Further Phase II study should be conducted to assessment these influence.

## Conclusion

Gamma knife radiosurgery or gefitinib when combined with WBRT increased the survival of NSCLC patients with brain metastases. The combination of GK and gefitinib following WBRT afforded the greatest survival benefit. Further study of therapies combined targeted therapies such as EGFR inhibitors and radiosurgery should be undertaken to identify brain metastasis patients most likely to benefit from aggressive, multimodality treatment. Further Phase II study should be conducted to assessment these influence.

## Abbreviation

WBRT: Whole brain irradiation
GK: Gamma knife radiosurgery
NHIRD: National Health Insurance Research Database
NSCLC: Non-small cell lung cancer
CCI: Charlson comorbidity index
EGFR: Epidermal growth factor receptor
TKI: Tyrosine kinase inhibitor
NCCN: National Comprehensive Cancer Network
SRS: Stereotactic radiosurgery
